# Supplementary material for: Zuo1 supports G4 structure formation and directs repair toward nucleotide excision repair
Source: Nat Commun. 2020 Aug 6;11:3907. doi: 10.1038/s41467-020-17701-8 (PMC7413387; doi:10.1038/s41467-020-17701-8)
Supplement: Supplementary file 1 — Supplementary Information [file 41467_2020_17701_MOESM1_ESM.pdf]

Supplementary Information

DeMagis et al

Zuo1 recognizes G4 structures and regulates the binding of repair factors

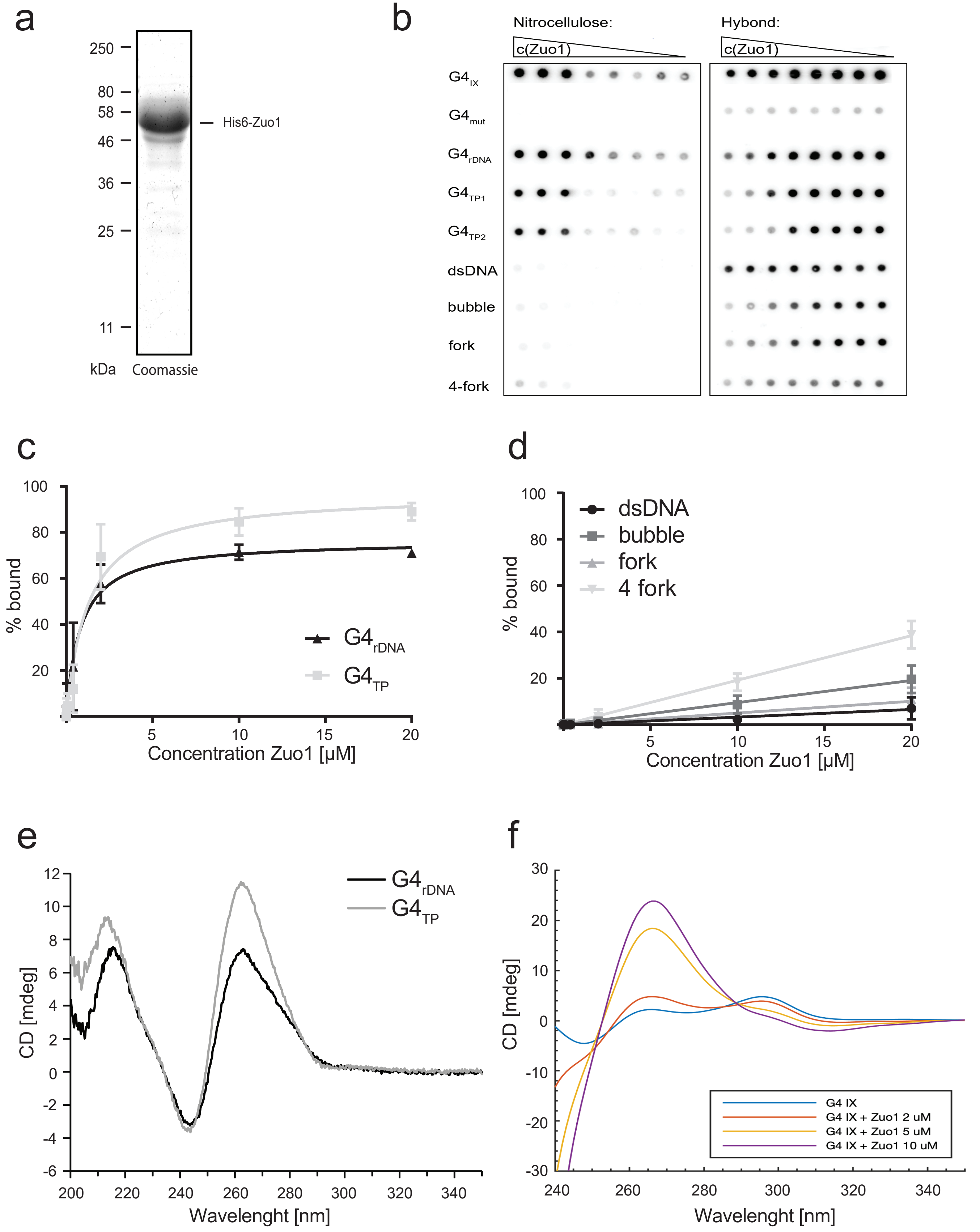

a
250
80
58
46
36
25
11
kDa
Coomassie
His6-Zuo1
b
Nitrocellulose:
Hybond:
c(Zuo1)
c(Zuo1)
G4IX
G4mut
G4rDNA
G4TP1
G4TP2
dsDNA
bubble
fork
4-fork
c
% bound
G4rDNA
G4TP
Concentration Zuo1 [μM]
d
dsDNA
bubble
fork
4 fork
% bound
Concentration Zuo1 [μM]
e
CD [mdeg]
G4rDNA
G4TP
Wavelenght [nm]
f
CD [mdeg]
G4 IX
G4 IX + Zuo1 2 uM
G4 IX + Zuo1 5 uM
G4 IX + Zuo1 10 uM
Wavelenght [nm]

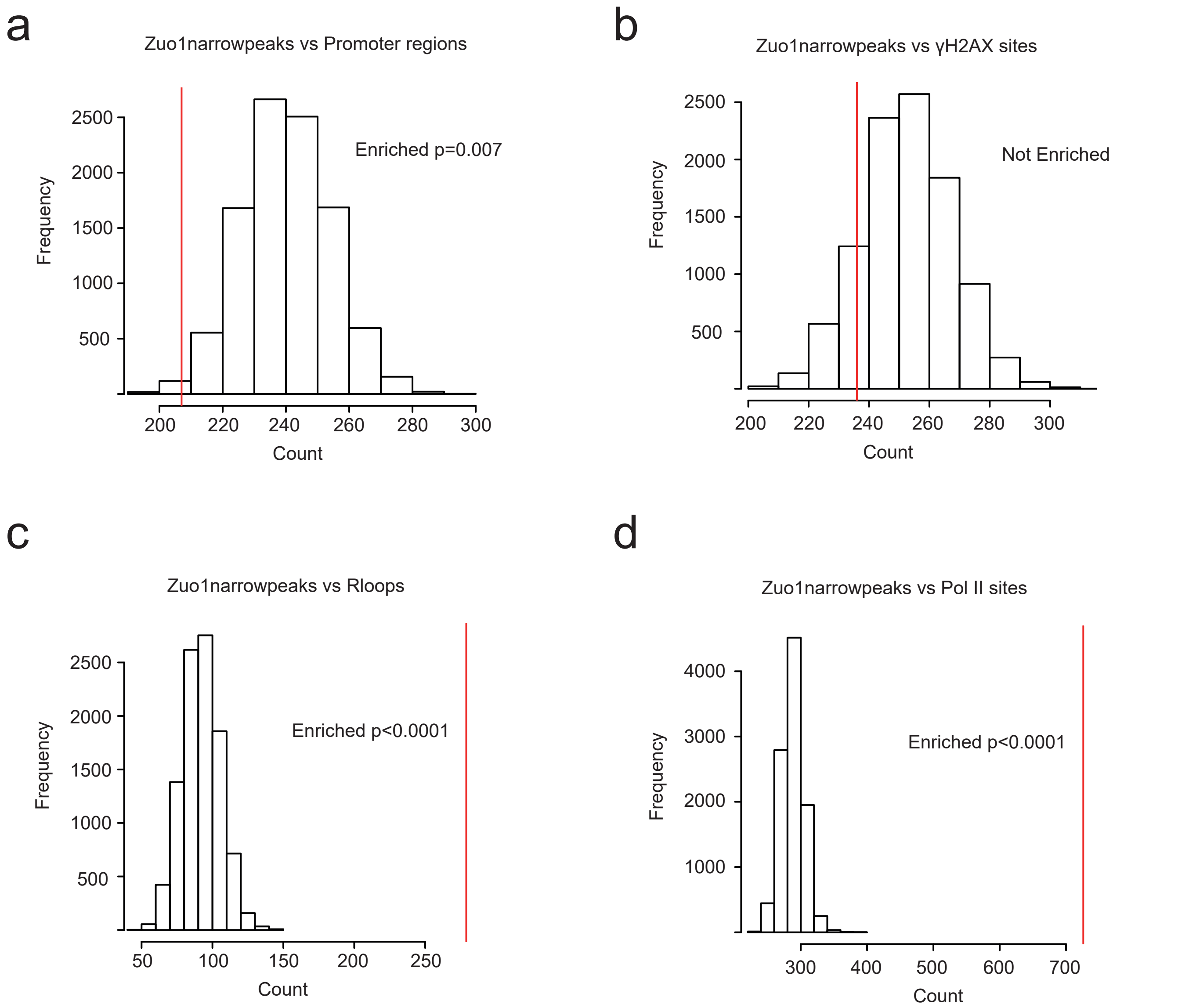

e

	Mean	SEM
WT	1	
zuo1Δ	0,555149	0,16645
Zuo1oex	0,972846	0,113404

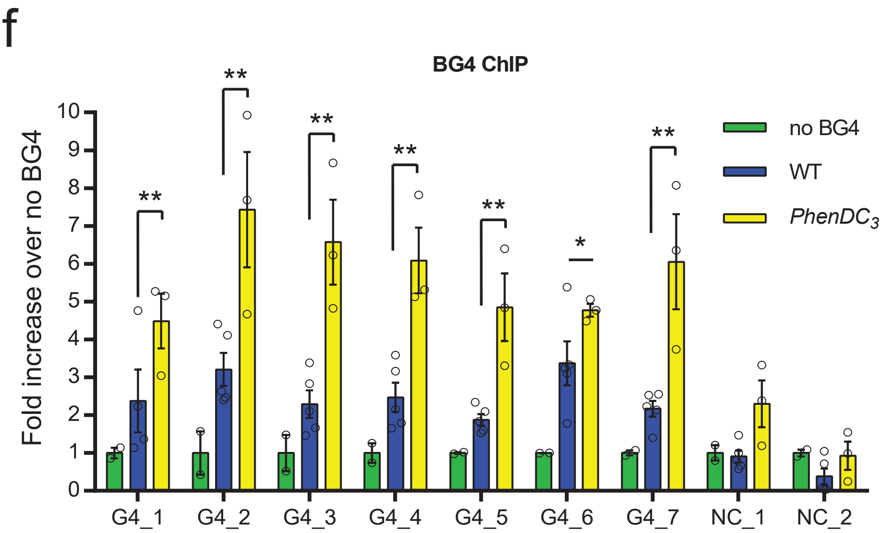

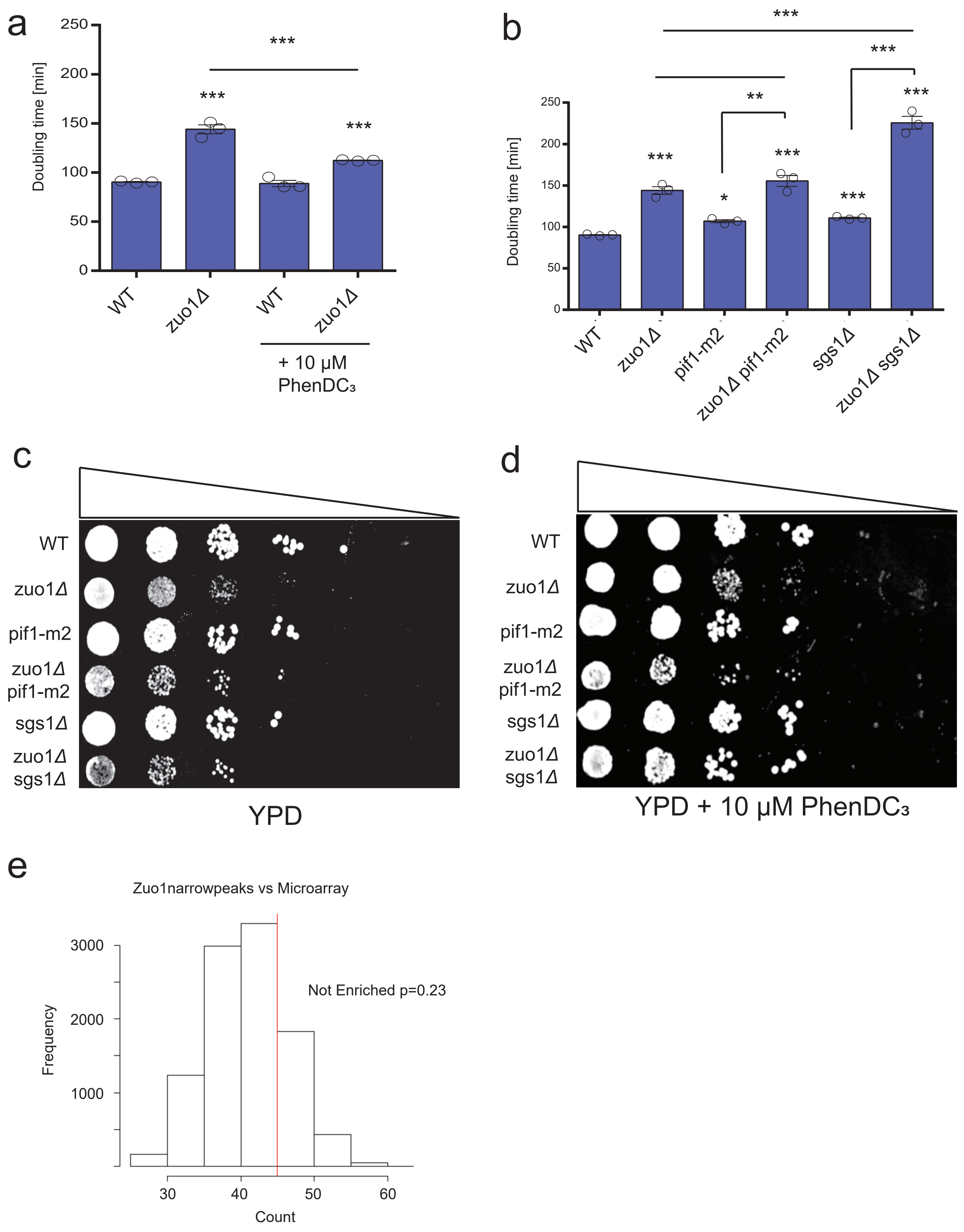
a
Doubling time [min]
250
200
150
100
50
0
***
***
***
WT
zuo1Δ
WT
zuo1Δ
+ 10 μM PhenDC₃
b
Doubling time [min]
250
200
150
100
50
0
***
***
**
***
***
*
***
***
WT
zuo1Δ
pif1-m2
zuo1Δ pif1-m2
sgs1Δ
zuo1Δ sgs1Δ
c
WT
zuo1Δ
pif1-m2
zuo1Δ pif1-m2
sgs1Δ
zuo1Δ sgs1Δ
YPD
d
WT
zuo1Δ
pif1-m2
zuo1Δ pif1-m2
sgs1Δ
zuo1Δ sgs1Δ
YPD + 10 μM PhenDC₃
e
Zuo1narrowpeaks vs Microarray
Frequency
3000
2000
1000
Not Enriched p=0.23
30
40
50
60
Count

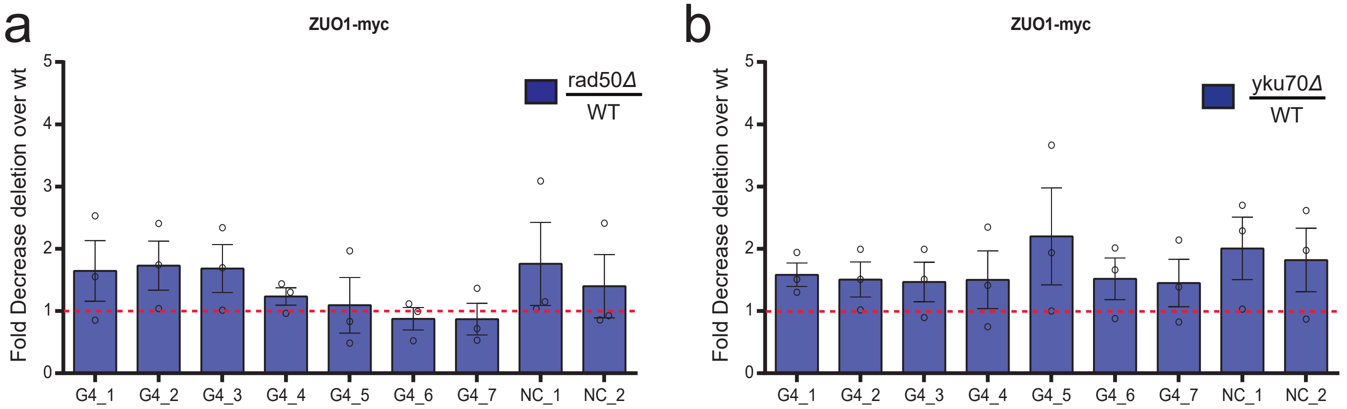

c

Strains	No Insertion	G4$_I$	G4mut
**WT**	1,0	1,1 ± 0,1	1,3 ± 0,04
**zuo1Δ**	0,7 ± 0,03	2,1 ± 0,3 *	1,9 ± 0,1 *

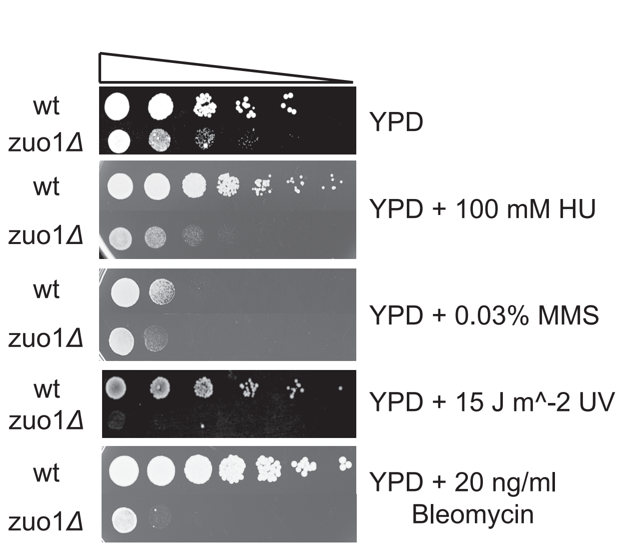

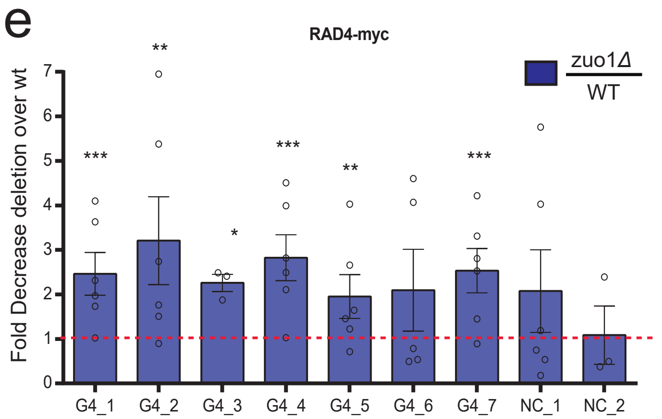

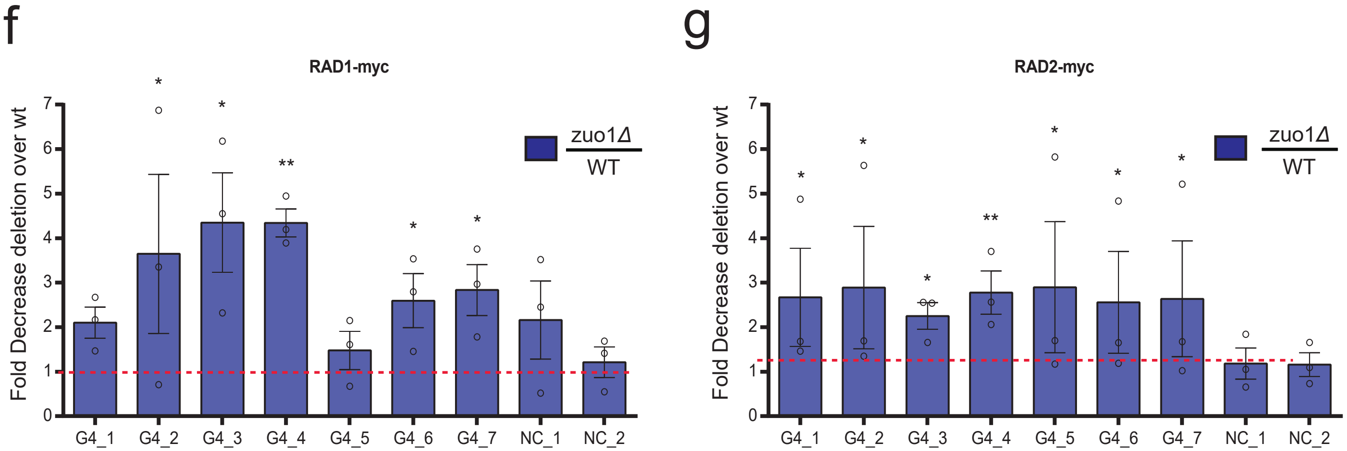

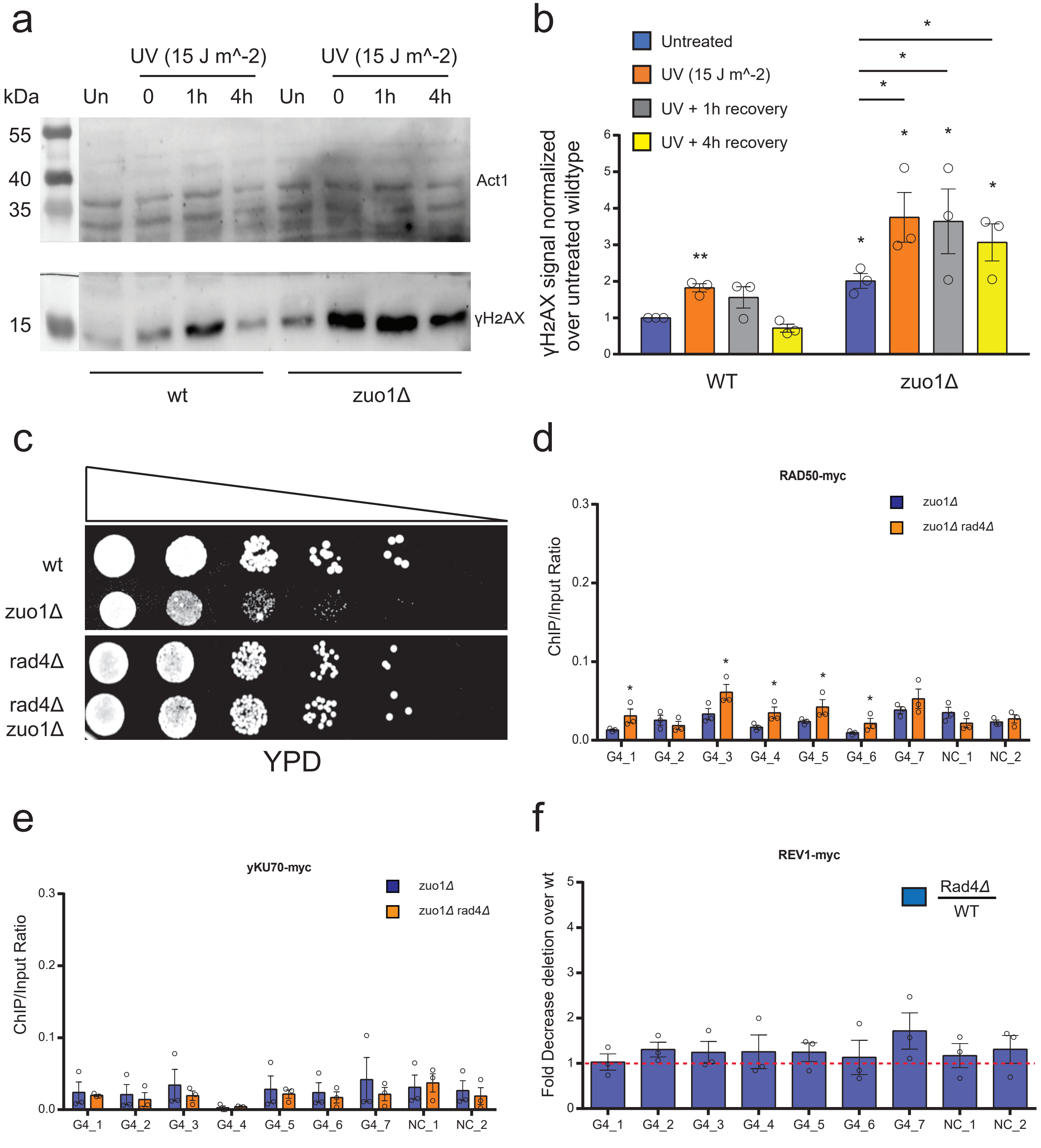
a
UV (15 J m^-2)
UV (15 J m^-2)
kDa
Un
0
1h
4h
Un
0
1h
4h
55
40
35
15
Act1
γH2AX
wt
zuo1Δ
b
Untreated
UV (15 J m^-2)
UV + 1h recovery
UV + 4h recovery
γH2AX signal normalized over untreated wildtype
WT
zuo1Δ
c
wt
zuo1Δ
rad4Δ
rad4Δ zuo1Δ
YPD
d
RAD50-myc
zuo1Δ
zuo1Δ rad4Δ
ChIP/Input Ratio
G4_1
G4_2
G4_3
G4_4
G4_5
G4_6
G4_7
NC_1
NC_2
e
yKU70-myc
zuo1Δ
zuo1Δ rad4Δ
ChIP/Input Ratio
G4_1
G4_2
G4_3
G4_4
G4_5
G4_6
G4_7
NC_1
NC_2
f
REV1-myc
Rad4Δ
WT
Fold Decrease deletion over wt
G4_1
G4_2
G4_3
G4_4
G4_5
G4_6
G4_7
NC_1
NC_2

a b

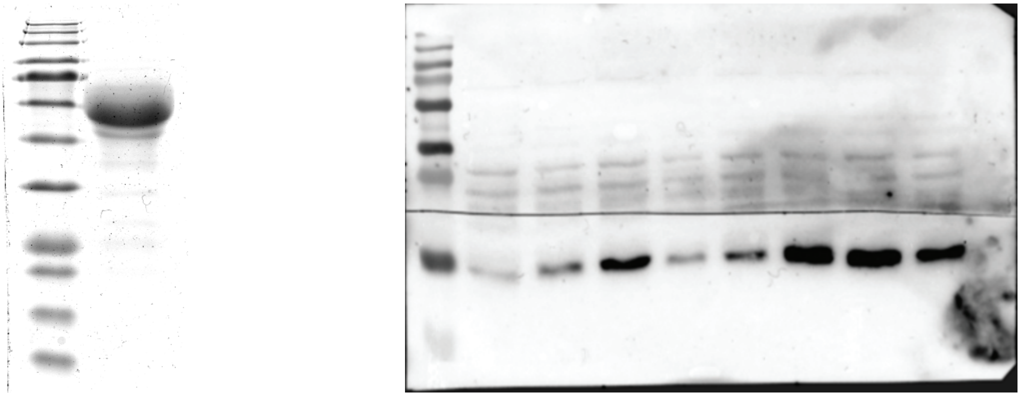

Supplementary Table 1: qPCR primers. The table shows the primers used for the qPCR analysis after ChIP experiments with myc or BG4 antibody. The first seven regions are selected by overlap between Zuo1 binding sites and G4 library. The last two are negative controls designed in G4 negative regions.

Name	Location	Orientation	Sequence
G4_1	XIIIb: G4 (Tract3)	Fw	GCTTCAGCCTGGGGTAAC
		Rv	GGCACCATTAGATTCACCAC
G4_2	XI: G4 (Tract3)	Fw	AATCCCGTCGCTATGCTC
		Rv	CTCCCGGTCTGTTATTTTC
G4_3	Ib: G4 (Tract2)	Fw	CCGATCCAGTCCCACAGTAA
		Rv	TGATCCTTGTCCCTTCCACC
G4_4	XV: G4 (Tract3)	Fw	ATACGCAGTATGGTGATATC
		Rv	GTTTATTGCCGATATACCTC
G4_5	X: G4 (Tract3)	Fw	CACAAACACATAAACACATAC
		Rv	CGGATTTCGCATAGTTGTC
G4_6	VII: G4 (Tract3, SOL4)	Fw	GACGTATCTTCATCGCTATC
		Rv	TATAATTACTTACAGTGCACATA
G4_7	II: G4 (Tract3, OM14)	Fw	CTCCTCTGTTCTTGCCTCA
		Rv	TACGCAGGGACGATTAACG
NC_1	ARO1: no G4	Fw	CTGCAGTCACAATTCCTCT
		Rv	TACGGATACCTAACCAGTC
NC_2	XIII: no G4	Fw	GAGGACGAAACGATTGATG
		Rv	AGATAATGAGCCACGGTAC

Supplementary Table 2: Yeast strains. The table shows the yeast strains used for all the assay performed. Those strains are generated by myc tagging or/and deletions of the S. cerevisiae standard laboratory strains W303 or Yph500.

Strain n°	Name	Genotype	Source
1	*Wildtype*	W303 MATa	R. Rothstein
2	*zuo1Δ*	MATa; zuo1::his	This paper
3	*pif1-m2*	MATa; zuo1::his	Paeschke et al. Cell
4	*sgs1Δ*	MATa; sgs1::his	Goetz et al. 2019
5	*zuo1Δ sgs1Δ*	MATa; zuo1::trp sgs1::his	This paper
6	*zuo1Δ pif1-m2*	MATa; zuo1::his	This paper
7	Sgs1myc	MATa; zuo1::his	This paper
8	Sgs1myc *zuo1Δ*	MATa; Sgs1-myc kan zuo1::his	This paper
9	Pif1myc	MATa; zuo1::his	Paeschke et al. Cell
10	Pif1myc *zuo1Δ*	MATa; Pif1-myc kan zuo1::his	This paper
11	Zuo1myc	MATa; Zuo1-myc trp	This paper
12	Zuo1myc *pif1-m2*	MATa; Zuo1-myc trp	This paper
13	Zuo1myc *sgs1Δ*	MATa; Zuo1-myc trp	This paper
14	Rad50myc	MATa; Rad50-myc trp zuo1::his	This paper
15	Rad50myc *zuo1Δ*	MATa; Rad50-myc trp zuo1::his	This paper
16	yKu70myc	MATa; yKu70-myc trp	This paper
17	yKu70myc *zuo1Δ*	MATa; yKu70-myc trp zuo1::his	This paper
18	Apn1myc	MATa; Apn1-myc trp	This paper
19	Apn1myc *zuo1Δ*	MATa; Apn1-myc trp zuo1::his	This paper
20	Rev1myc	MATa; Rev1-myc trp	This paper
21	Rev1myc *zuo1Δ*	MATa; Rev1-myc trp zuo1::his	This paper
22	Rad23myc	MATa; Rad23-myc trp	This paper
23	Rad23myc *zuo1Δ*	MATa; Rad23-myc trp zuo1::his	This paper
24	Rad4myc	MATa; Rad4-myc trp	This paper
25	Rad4myc *zuo1Δ*	MATa; Rad4-myc trp zuo1::his	This paper
26	Zuo1myc *rad50Δ*	MATa; Zuo1-myc trp rad50::his	This paper
27	Zuo1myc *yku70Δ*	MATa; Zuo1-myc trp yku70::his	This paper
28	Rad1myc	MATa; Rad1-myc trp	This paper
29	Rad1myc *zuo1Δ*	MATa; Rad1-myc trp zuo1::his	This paper
30	Rad2myc	MATa; Rad2-myc trp	This paper
31	Rad2myc *zuo1Δ*	MATa; Rad2-myc trp zuo1::his	This paper
32	*rad4Δ*	MATa; rad4::kan	This paper
33	*zuo1Δ rad4Δ*	MATa; zuo1::his rad4::kan	This paper
34	yKu70myc *zuo1Δ rad4Δ*	MATa; zuo1::his	This paper
35	Rad50myc *zuo1Δ rad4Δ*	MATa; zuo1::his	This paper
36	Rev1myc *zuo1Δ rad4Δ*	MATa; zuo1::his	This paper
37	Rev1myc *rad4Δ*	MATa; zuo1::his	This paper

Supplementary Table 3. Binding assay´s sequence. The table shows the sequence used for the binding assay analysis.

G4 motif	*S.cerevisiae* chromosome	Sequence 5’-3’
$G4_{rDNA}$	XII	**GGG**TAAC**GGGG**AATAA**GGG**TTCGATTCCGGAGA**GGG**
$G4_{TP}$	N/A	**GGGGG**AGCT**GGGG**TAGAT**GGG**AATGTGA**GGG**
$G4_{ChrIX}$	IX	**GGG**TACGGT**GGG**TAATAA**GGG**AAGGTATC**GGG**
$G4_{mut}$	N/A	**GCG**TACGGT**GCG**TAATAA**CGC**AAGCTATC**GCG**
dsDNA	N/A	GACGCTGCCGAATTCTGGCTTGCTAGGACATCTTTGCCCAC GTTGACCCG + CGGGTCAACGTGGGCAAAGATGTCCTAGCAAGCCAGAATT CGGCAGCGTC
Bubble	N/A	CGGGTCAACGTGGGCAAAGCCAATGCGATCGGCCAGAATT CGGCAGCGTC + GACGCTGCCGAATTCTGGCTTGCTCGGACATCTTTGCCCAC GTTGACCCG
forked dsDNA	N/A	GACGCTGCCGAATTCTGGCTTGCTAGGACATCTTTGCCCAC GTTGACCCG + CGGGTCAACGTGGGCAAAGATGTCCTAGCAATGTAATCGT CTATGACGTC
4 fork	N/A	GACGCTGCCGAATTCTGGCTTGCTAGGACATCTTTGCCCAC GTTGACCCG + CGGGTCAACGTGGGCAAAGATGTCCTAGCAATGTAATCGT CTATGACGTC + GACGTCATAGACGATTACATTGCTAGGACATGCTGTCTAGA GACTATCGC + GCGATAGTCTCTAGACAGCATGTCCTAGCAAGCCAGAATTC GGCAGCGTC

Supplementary Figures

Fig. S1 Zuo1, an *in vitro* G4-binding protein. **a)** Coomassie staining of purified Zuo1. 6xHis-tagged Zuo1 (52 kDa) Purification was reproducible in three independent approaches **b)** Double-filter binding assays were performed with purified Zuo1 and different DNA structures. Autoradiography visualized the Zuo1-G4 interaction on the nitrocellulose membrane, whereas unbound DNA is visible on the Hybond membrane. **c)** Quantification of Zuo1 binding to $G4_{IX}$ and $G4_{mut}$ by filter binding assay. Error bars correspond to one standard deviation of the mean of 3 independent experiments. **d)** Quantification of Zuo1 binding to dsDNA, bubble, fork and 4-fork by filter binding assay. Error bars correspond to one standard deviation of the mean of 3 independent experiments. **e)** CD spectra of folded oligonucleotides $G4_{rDNA}$ and $G4_{TP}$ in the presence of 100 mM K^+. **f)** CD spectra of folded oligonucleotides $G4_{IX}$ in the presence of different concentrations of Zuo1 (2, 5, 10 µM).

Fig. S2 Zuo1 recognizes and stabilizes G4s *in vivo*. **a)** Genome-wide correlation of the Zuo1 peaks with the promoter regions library. **b)** Genome wide correlation of the Zuo1 peaks with the regions that are high in γH2AX. **c)** Genome-wide correlation of the Zuo1 peaks with the R-loop structures. **d)** Genome-wide correlation of the Zuo1 peaks with the genome-wide peaks of DNA Pol II sites. All Genome wide peaks are peaks are obtained based on three biological replicates **e)** Quantification of the BG4 filter assay. **f)** BG4-ChIP analysis followed by qPCR of G4 levels in wildtype (blue), no tag (green) and wildtype + 10 µM $PhenDC_3$ (yellow). The bars show the G4 levels of n=3 biologically independent experiments for wildtype and wildtype + 10 µM $PhenDC_3$ normalized over the no-tag strain (n=2 biologically independent experiments) ± SEM. Significance was calculated based on one-sided Student's t-test. Asterisks indicate statistical significance in comparison with wildtype cells. *p < 0.05, **p < 0.01, ***p < 0.001, ****p < 0.0001.

Fig. S3 Zuo1 function at G4 is linked to helicase action. a,b) Growth analysis in liquid media, doubling times (minutes) of n=3 biologically independent experiments were calculated using indicated yeast strains. Error bars present ± SEM. Significance was calculated based on one-sided Student's t-test. Asterisks indicate statistical significance: *p < 0.05, **p < 0.01, ***p < 0.001. **a)** Rich media plus the addition of 10 µM of $PhenDC_3$ **b)** complete media (YPD). **c,d)** Different cell numbers of yeasts were spotted on media in a serial dilution. Growth changes were monitored by colonies formation. **c)** complete media (YPD) **d)** complete media (YPD) plus the addition of 10 µM of $PhenDC_3$ **e)** Genome-

wide correlation of the Zuo1 peaks with the up- and down-regulated genes obtained by microarray analysis.

Fig. S4 Zuo1 drive NER pathway recognition at G4 sites. a,b) Zuo1-Myc-ChIP analysis followed by qPCR of Zuo1 binding at 9 different loci. Decrease in binding of n=3 biologically independent experiments is plotted. Data are normalized over the wildtype strain ± SEM. **a)** Zuo1 binding in Rad50 deletion (*rad50Δ*). **b)** Zuo1 binding in yKu70 deletion (*ku70Δ)* **c)** Quantification of the GCR assay. Plotted results were based on the average of, at least, three independent experiments. **d)** Yeast cells were spotted, at different concentrations (serial dilution) on different plates, including YEPD and different DNA damaging conditions (HU, MMS, Bleomycin and UV treatment), concentrations of drugs and treatment are indicated in the Figure. Growth changes and sensitivity were monitored by colonies formation. **e-g)** Binding of NER proteins by ChIP and qPCR analysis in wildtype and *zuo1Δ* cells. All qPCRs were performed at seven Zuo1 targets and two controls. Presented data show a decrease in binding of n=3 biologically independent experiments due to the normalization: *zuo1* over wildtype strain ± SEM. Significance was calculated based on one-sided Student's t-test. Asterisks indicate statistical significance in comparison to wildtype cells. *p < 0.05, **p < 0.01, ***p < 0.001, ****p < 0.0001. **e)** ChIP and qPCR of Rad4-Myc. **f)** ChIP and qPCR of Rad1-Myc **g)** ChIP and qPCR of Rad2-Myc Significance was calculated based on the Student's t-test. Asterisks indicate statistical significance in comparison with wildtype cells. *p < 0.05, **p < 0.01, ***p < 0.001, ****p < 0.0001. All plotted results were based on the average of, at least, three independent experiments.

Fig. S5 Increase of TLS activity rescue the Zuo1 growth defects in the double mutant zuo1Δ *rad4Δ*. a) Western Blot analysis of protein extract from wildtype and *zuo1Δ* untreated or treated with UV light and stained with γH2AX and Act1 antibodies. The treated samples are extracted directly after UV light treatment or after 1-hour recovery (1 h lanes) and 4-hour recovery (2 h lanes) in normal YPD media. Similar results were obtained in three independent experiments **b)** Western Blot quantification, values of n=3 biologically independent experiments are normalized over untreated wildtype. Significance was calculated based on one-sided Student's t-test. Asterisks indicate statistical significance: *p < 0.05, **p < 0.01, ***p < 0.001. **c)** Different numbers of yeast cells were spotted on rich media in a serial dilution. Growth changes were monitored by colonies formation. **d,e**) ChIP and qPCR analysis of different repair proteins, in *zuo1Δ* (blue) compare with the double mutant *zuo1Δ rad4Δ* (orange). The bars show the IP value over input ± SEM of n=3 biologically independent experiments.

All qPCRs were performed at seven Zuo1 targets and two controls. **d)** ChIP and qPCR of Rad50-Myc. Significance was calculated based on one-sided Student's t-test. Asterisks indicate statistical significance: *p < 0.05, **p < 0.01, ***p < 0.001. **e)** ChIP and qPCR of yKU70-Myc. **f)** Rev1 Myc-ChIP analysis followed by qPCR to monitor Rev1 binding to 9 different loci. The bars show the Rad1 binding decrease of n=3 biologically independent experiments of the *rad4Δ* deletion normalized over the wildtype strain ± SEM. Significance was calculated based on the Student's t-test. Asterisks indicate statistical significance in comparison with wildtype cells. *p < 0.05, **p < 0.01, ***p < 0.001, ****p < 0.0001. All plotted results were based on the average of, at least, three independent experiments.

Fig. S6 Full scan images a) Full scan images of Coomassie stained gels after Zuo1 purification (see Supplementary Figure S1a) **b)** Full scan gel image of γH2AX Western Blot from Supplementary Figure S5a
