# Supplementary material for: Zuo1 supports G4 structure formation and directs repair toward nucleotide excision repair
Source: Nat Commun. 2020 Aug 6;11:3907. doi: 10.1038/s41467-020-17701-8 (PMC7413387; doi:10.1038/s41467-020-17701-8)
Supplement: Supplementary file 3 — Description of Additional Supplementary Files [file 41467_2020_17701_MOESM3_ESM.pdf]

Description of Additional Supplementary Files

Supplementary data 1: After Y1H screen, plasmids where re-isolated, re-cloned and sequenced. BLAST analysis of sequencing read of interacting proteins are listed below. Proteins are listed in alphabetical order. Numbers in the second column indicates how often we identified the peak. Zuo1 was identified 18x in Y1H screen. Y1H screen was performed in pif1-m2 strains, to enhance G4 formation in the assay. Ribosomale or proteins involved in translation are highlighted in grey.

Supplementary data 2: Zuo1 peaks obtained by ChIP performed with myc antibody followed by deep sequencing. The peaks were called by MACS 2.0 using standard settings. The following information are provided: chromosome, start and end coordinates, length and fold enrichment over input.

Supplementary data 3: Genes up and down regulate in zuo1Δ compared with wildtype strain.
