# Supplementary material for: Zuo1 supports G4 structure formation and directs repair toward nucleotide excision repair
Source: Nat Commun. 2020 Aug 6;11:3907. doi: 10.1038/s41467-020-17701-8 (PMC7413387; doi:10.1038/s41467-020-17701-8)
Supplement: Supplementary file 7 — Reporting Summary [file 41467_2020_17701_MOESM7_ESM.pdf]

nature research

Corresponding author(s): Katrin Paeschke

Last updated by author(s): Jun 22, 2020

Reporting Summary

Nature Research wishes to improve the reproducibility of the work that we publish. This form provides structure for consistency and transparency in reporting. For further information on Nature Research policies, see our Editorial Policies and the Editorial Policy Checklist.

Statistics

For all statistical analyses, confirm that the following items are present in the figure legend, table legend, main text, or Methods section.

n/a	Confirmed	
☐	☒	The exact sample size (n) for each experimental group/condition, given as a discrete number and unit of measurement
☐	☒	A statement on whether measurements were taken from distinct samples or whether the same sample was measured repeatedly
☐	☒	The statistical test(s) used AND whether they are one- or two-sided<br>*Only common tests should be described solely by name; describe more complex techniques in the Methods section.*
☐	☒	A description of all covariates tested
☐	☒	A description of any assumptions or corrections, such as tests of normality and adjustment for multiple comparisons
☐	☒	A full description of the statistical parameters including central tendency (e.g. means) or other basic estimates (e.g. regression coefficient) AND variation (e.g. standard deviation) or associated estimates of uncertainty (e.g. confidence intervals)
☐	☒	For null hypothesis testing, the test statistic (e.g. F, t, r) with confidence intervals, effect sizes, degrees of freedom and P value noted<br>*Give P values as exact values whenever suitable.*
☒	☐	For Bayesian analysis, information on the choice of priors and Markov chain Monte Carlo settings
☒	☐	For hierarchical and complex designs, identification of the appropriate level for tests and full reporting of outcomes
☒	☐	Estimates of effect sizes (e.g. Cohen's d, Pearson's r), indicating how they were calculated

Our web collection on statistics for biologists contains articles on many of the points above.

Software and code

Policy information about availability of computer code

Data collection	The following commerical software was used to collect data: MACS 2.0, bowtie
Data analysis	The following commerical software was used to analyse data: Microsoft Excel (latest release), ImageJ (latest release), GraphPad Prism 6

For manuscripts utilizing custom algorithms or software that are central to the research but not yet described in published literature, software must be made available to editors and reviewers. We strongly encourage code deposition in a community repository (e.g. GitHub). See the Nature Research guidelines for submitting code & software for further information.

Data

Policy information about availability of data

All manuscripts must include a data availability statement. This statement should provide the following information, where applicable:
- Accession codes, unique identifiers, or web links for publicly available datasets
- A list of figures that have associated raw data
- A description of any restrictions on data availability

The data that support the findings of this study are available via the Accession code given in the manuscript

Field-specific reporting

Life sciences study design

All studies must disclose on these points even when the disclosure is negative.

Sample size	A minimum of Biological Triplicate is used for each analysis. We have chosen 3 biological replicates to increase statistical significances, no sample size calculation was performed. Sample sizes were chosen according to our previous experience in similar experimental setups to generate tatistically significant publication quality values
Data exclusions	No data were excluded from the analysis
Replication	All experiments were repeated in biological triplicates to ensure the reproducibility of the experiment. The number of replicates for each figure is clearly stated in the figure legend
Randomization	randomazation was not required
Blinding	Blinding was not required

Reporting for specific materials, systems and methods

We require information from authors about some types of materials, experimental systems and methods used in many studies. Here, indicate whether each material, system or method listed is relevant to your study. If you are not sure if a list item applies to your research, read the appropriate section before selecting a response.

Materials & experimental systems

n/a	Involved in the study	
☐	☒	Antibodies
☐	☒	Eukaryotic cell lines
☒	☐	Palaeontology and archaeology
☒	☐	Animals and other organisms
☒	☐	Human research participants
☒	☐	Clinical data
☒	☐	Dual use research of concern

Methods

n/a	Involved in the study	
☐	☒	ChIP-seq
☒	☐	Flow cytometry
☒	☐	MRI-based neuroimaging

Antibodies

Antibodies used	Anti-c-myc (Takara cat#631206), FLAG M2 Magnetic Beads (Sigma cat#M8823), Anti-γH2AX (Abcam cat#Ab15083), Anti-Act1 (Santa Cruz Biotechnology cat#SC398161), Anti-HRP (Santa Cruz Biotechnology cat#SC2031, cat#SC2357) BG4 antibody (Homemade Expression)
Validation	The anti-Myc antibody was validated in previous publication (e.g. Wanzek et al. doi:10.1093/nar/gkx467, Paeschke et al. doi:10.1016/j.cell.2011.04.015). Astrain lacking the Myc-tag was used as a negative control of the Myc-ChIP analysis. BG4 antibody purity was monitored by SDS-PAGE after each preparation. Affinity for G4 structures was checked by IF and ChIP. As positive control cells were treated with well-known G4 stabilizers (phen-DC3). Anti-γH2AX (Abcam cat#Ab15083), Anti-Act1 (Santa Cruz Biotechnology cat#SC398161), Anti-HRP (Santa Cruz Biotechnology cat#SC2031, cat#SC2357) BG4 antibody (Homemade Expression) have been validated by their respective manufactures.

Eukaryotic cell lines

Policy information about cell lines

Cell line source(s)	All the strains used in this work are derivatives of the RAD5+ version of W303 (R. Rothstein) or YPH background
Authentication	*Describe the authentication procedures for each cell line used OR declare that none of the cell lines used were authenticated.*
Mycoplasma contamination	N/A
Commonly misidentified lines (See ICLAC register)	N/A

ChIP-seq

Data deposition

- [x] Confirm that both raw and final processed data have been deposited in a public database such as GEO.
- [x] Confirm that you have deposited or provided access to graph files (e.g. BED files) for the called peaks.

Data access links<br>*May remain private before publication.*	https://www.ncbi.nlm.nih.gov/geo/query/acc.cgi?acc=GSE149502
Files in database submission	Bam files of the ChIP-seq experiment and file with the peaks calles by MACS2
Genome browser session<br>(e.g. UCSC)	https://genome-euro.ucsc.edu/s/stanmiju/DeMagis_2020

Methodology

Replicates	ChIP-seq was performed as a duplicate
Sequencing depth	All reads are 64 bp long and single end. DeMagis_Zuo_IP_1.bam, 3143419,reads, DeMagis_Zuo_input_1.bam,32810705,reads, DeMagis_Zuo_IP_2.bam,2962173,reads, DeMagis_Zuo_input_2.bam,19531001,reads
Antibodies	anti-Myc antibody (Takara), BG4 antibody (Homemade Expression)
Peak calling parameters	callpeak -t DeMagis_Zuo_IP_1.bam DeMagis_Zuo_input_1.bam DeMagis_Zuo_IP_2.bam -c DeMagis_Zuo_input_1.bam DeMagis_Zuo_IP_2.bam DeMagis_Zuo_input_2.bam -n zuo1-q 0.05 -g ce
Data quality	200 peaks are enriched more than 5-fold. Fastq files were controlled by fastqc. Peaks were checked via qPCR.
Software	MACS2, bowtie2 and fastQC was used
